# Cardiac Involvement in COVID-19: A Global Bibliometric and Visualized Analysis

**DOI:** 10.3389/fcvm.2022.955237

**Published:** 2022-07-27

**Authors:** Si-chi Xu, Xin-yue Zhao, Hai-ping Xing, Wei Wu, Shu-yang Zhang

**Affiliations:** State Key Laboratory of Complex Severe and Rare Diseases, Department of Cardiology, Peking Union Medical College Hospital, Chinese Academy of Medical Science and Peking Union Medical College, Beijing, China

**Keywords:** COVID-19, cardiac, heart, bibliometric, myocarditis, heart failure

## Abstract

**Objective:**

Coronavirus disease 2019 (COVID-19), which was caused by severe acute respiratory syndrome coronavirus-2 (SARS-CoV-2), had already resulted in widespread epidemics worldwide and millions of people's deaths since its outbreak in 2019. COVID-19 had also been demonstrated to affect people's cardiac function. However, the specific mechanism and influence of this damage were not clear yet. The purpose of the present study was to provide a bibliometric analysis of the current studies related to cardiac involvement after SARS-CoV-2 infection.

**Methods:**

A bibliometric literature search was performed on the web of science. The number and type of publications, countries, institutional sources, journals, and citation patterns were analyzed. In addition, qualitative and quantitative evaluations were carried out to visualize the scientific achievements in this field by using the VOSviewer software.

**Results:**

Web of science had recorded 2,24,097 documents on COVID-19 at the time of data collection (May 12, 2022). A total of 2,025 documents related to cardiac involvement were recorded at last. The countries with the most published articles were the United States of America (USA) (*n* =747, 36.9%), Italy (*n* =324, 16%), and England (*n* =213, 10.5%). Although the countries and institutions that published the most articles were mainly from the USA, the top three authors were from Germany, England, and Poland. Frontiers in Cardiovascular Medicine was the journal with the most studies (65 3.2%), followed by ESC Heart Failure (59 2.9%) and Journal of Clinical Medicine (56 2.8%). We identified 13,739 authors, among which Karin Klingel and Amer Harky had the most articles, and Shaobo Shi was co-cited most often. There existed some cooperation between different authors, but the scope was limited. Myocarditis and heart failure (HF) were the main research hotspots of COVID-19 on cardiac dysfunction and may be crucial to the prognosis of patients.

**Conclusions:**

It was the first bibliometric analysis of publications related to COVID-19-associated cardiac disorder. This study provided academics and researchers with useful information on the most influential articles of COVID-19 and cardiac dysfunction. Cooperation between countries and institutions must be strengthened on myocarditis and HF during COVID-19 pandemic.

## Introduction

Coronavirus disease 2019 (COVID-19), which leads to a global pandemic, is caused by severe acute respiratory syndrome coronavirus 2 (SARS-CoV-2). Up to now, COVID-19 has caused millions of deaths, which resulted in a catastrophic impact on global systems. COVID-19 mainly attacks the respiratory system. The most common symptoms in the early period are fever, dry cough, and shortness of breath (1). With the increase in the number of infected people, more and more clinical evidence showed that COVID-19 had a serious effect on various systems and multiple organ injuries in severe patients aggravated the difficulty of treatment (2). Previous studies also suggested that COVID-19 patients with cardiovascular disease were often more severely ill and had a higher risk of death, especially in elderly patients (3). In addition, doctors from many countries had reported that compared with the general population, patients with cardiovascular disease had a worse prognosis for SARS-CoV-2 infection. Early literature reported that COVID-19 can directly or indirectly cause a series of cardiovascular damage, ranging from acute myocardial injury, myocarditis, cardiomyopathy, acute coronary syndrome, and myocardial infarction (4). It can be suggested that COVID-19 and cardiovascular diseases affect each other and together lead to acute and malignant adverse events. Therefore, it is particularly vital to pay special attention to the pathological characteristics, clinical manifestations, disease process, and prognosis of cardiac damage caused by COVID-19.

Bibliometrics was first proposed by American bibliographers in 1969. Bibliometrics belongs to the discipline that applies mathematical and statistical methods to the study of books or other communication media (5). A bibliometric study is aimed to introduce this topic uniquely and comprehensively and provide evidence-based practice. Bibliometrics major in related papers of a specific field by statistical analysis and the results can reflect the research status of a specific topic, including main countries, research institutions/organizations, researchers, and the main journals that published related literature. As the pandemic continued in the world, bibliometric assessments on a wide range of issues were published in COVID-19 (6, 7). Although there were many reports on the cardiovascular disorder caused by COVID-19, the previous literature had not systematically described and summarized the effect yet. At the same time, as the epidemic situation had a serious impact on daily communication, it was necessary to summarize the experience due to the different epidemic prevention policies taken by the various countries and regions. Although vaccines are available, we still need 70% to 80% of the population with active immunity through infection or vaccines to cut down the disease chain. Therefore, bibliometric analysis was adopted to guide future research priorities by evaluating the most relevant scientific research on COVID-19 and cardiac dysfunction. This study significantly contribute to the allocation and refinement of future cardiac research caused by COVID-19.

## Methods

### Study Design

Bibliometric techniques were used to perform a descriptive cross-sectional analysis of publications relevant to cardiac involvement in COVID-19.

### Database Used

SCI-E and SSCI of the core database of the document information index database Web of Science (WOS) were selected for source document retrieval. The search formula was set to TS = (“cardiac” OR “heart” OR “cardiomyocyte”) AND TS = (“SARS-CoV-2” OR “COVID-19” OR “2019-nCoV”), and the literature search time of the present study was from the earliest time of publication in the database to the latest time of literature publication (May 12, 2022).

### Data Analysis

VOSviewer was used to analyze the exported articles. VOSviewer displayed a map based on the construction of the co-occurrence matrix. The similarity matrix was calculated to refer to the co-occurrence matrix and the map was visualized by a special VOS mapping technique. The term co-occurrence graph in VOSviewer only includes terms that appear in the title and abstract at least 50 times under the binary count (8). The algorithm can make it possible that the terms occur more frequently entitled with larger bubble images and that terms with high similarity are close to each other (9).

## Results

### General Description of the Retrieved Publications

**Figure 1:**
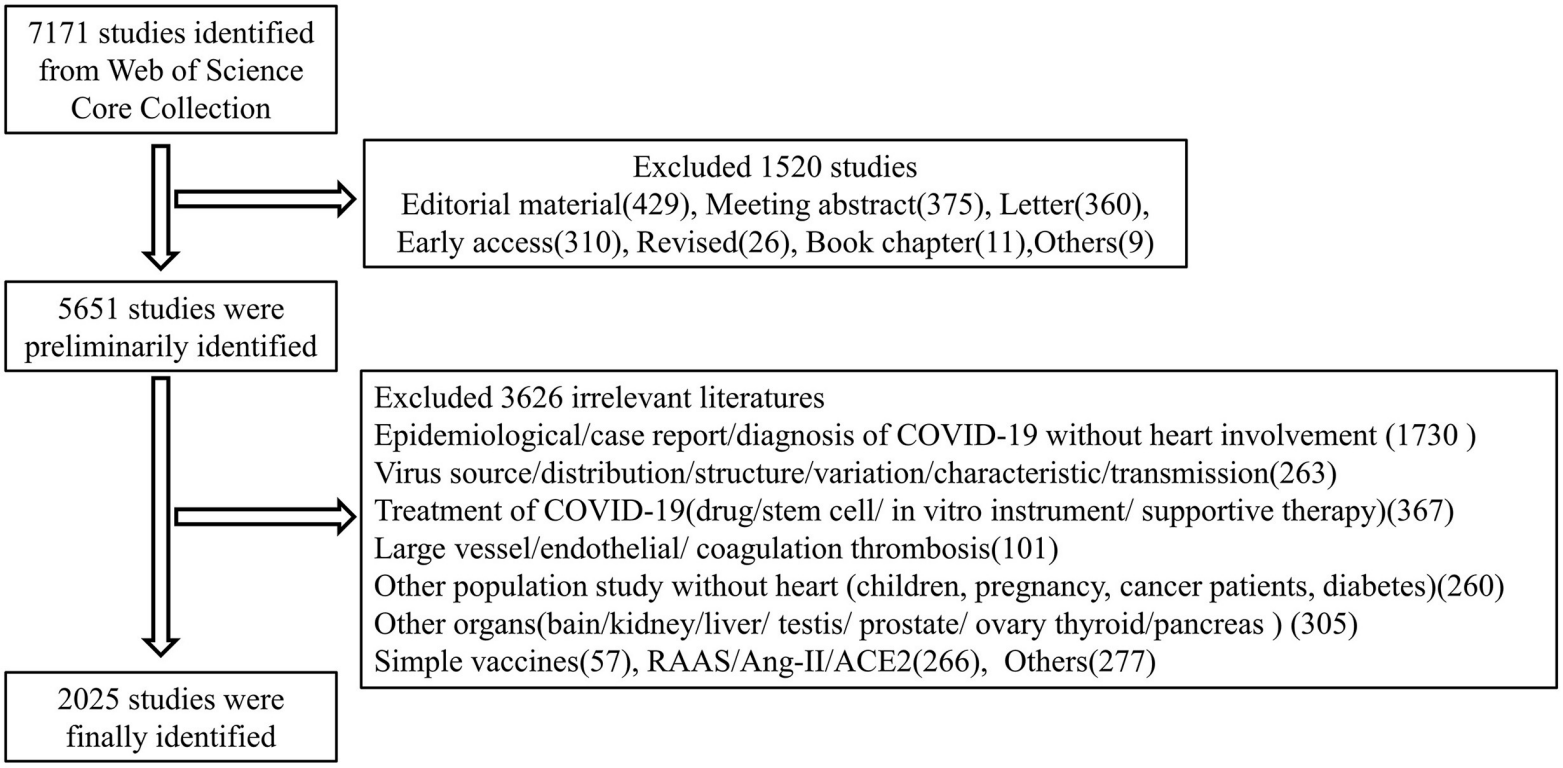
The detailed information of the screening process. All of the relevant studies of COVID-19 and heart were primarily recorded by the initial retrieval in the web of science. Only articles and reviews were selected for the second analysis. We eliminated invalid documents of which the themes were not related to COVID-19 and heart. Finally, a total of 2025 records were used as the dataset in the final study.

Web of science has published 2,24,097 documents on COVID-19 in all study fields at the time of data collection [TS=(“SARS-CoV-2” OR “COVID-19” OR “2019-nCoV”)]. A total of 7,171 documents related to cardiac involvement were primarily retrieved by the corresponding mesh terms. Of these, 4,300 (60%) were research articles, 1,351 (18.8%) were reviews, 429 (6%) were editorial materials, and 375 (5.2%) were meeting abstracts. Detailed information of the articles was presented in Table 1. To obtain more accurate information about the impact of COVID-19 on the heart, we further screened all of the included literature by the title and abstract. A total of 3,626 articles were removed without specific cardiac involvement and 2,025 studies were included in the final analysis. The detailed information of the screening process was shown in Figure 1.

**Table 1:** Summary of all literature initially included in the study.

**Publication type**	**Counts**	**%**
Article	4,300	60.0
Review	1,351	18.8
Editorial material	429	6.0
Meeting abstract	375	5.2
Letter	360	5.0
Early access	310	4.3
Revised	26	0.4
Book Chapter	11	0.2
News item/Conference papers/Retraction/Data paper	9	0.1

### Distribution of Countries

**Figure 2:**
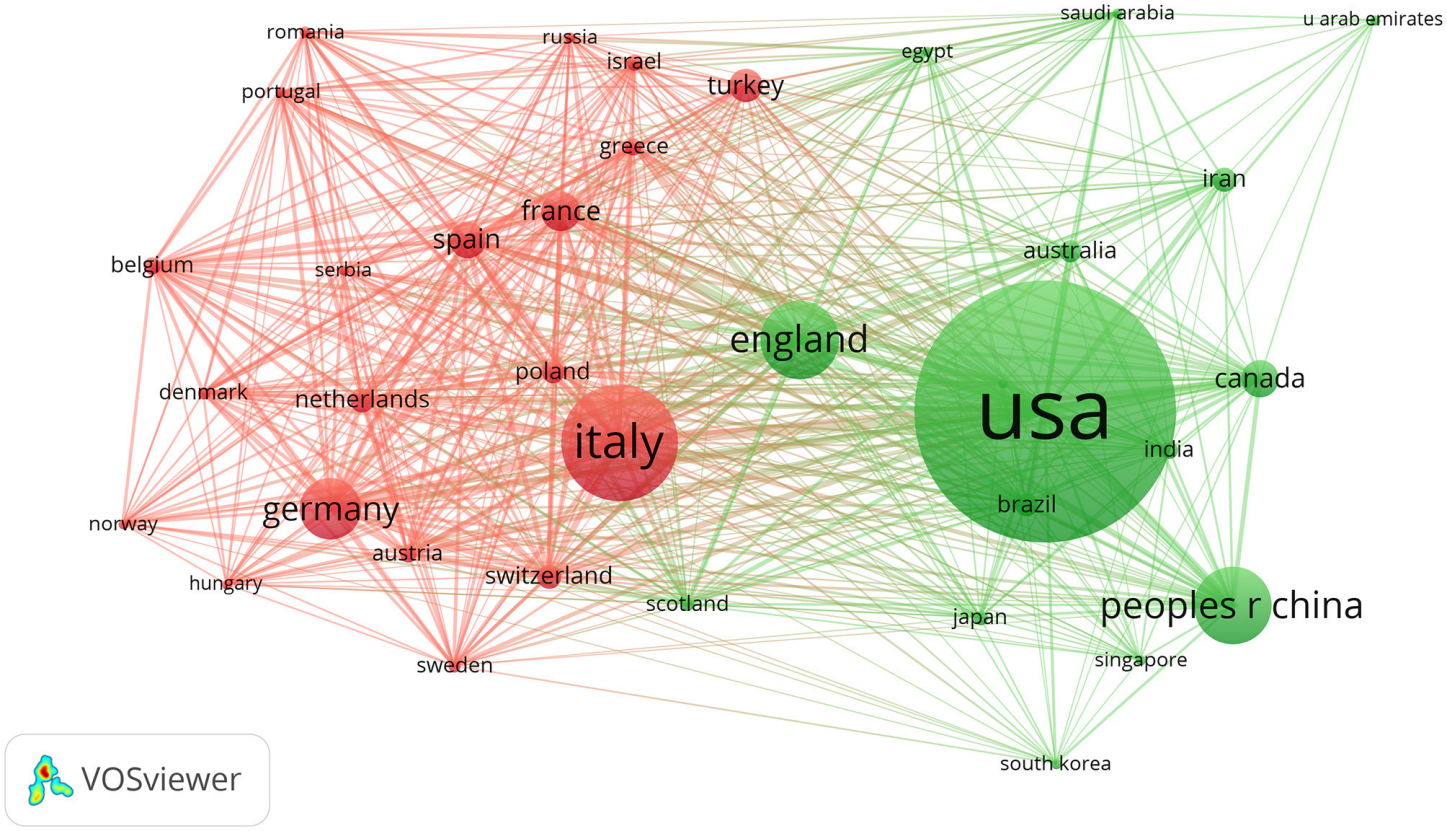
The detailed information of country distribution. The terms were represented by nodes, while links (lines) connected the nodes. The size of the circle was proportional to its number of publications, while the width of the line between the two items was related to the magnitude of their collaboration. Items of the same color belonged to the same cluster, indicating that they cooperated closely in this field. The more clusters there were, the more decentralized the cooperation was. The USA, Italy, and England were the top three countries that published the most articles. They were mainly divided into two clusters as shown in this figure. Cluster 1 included the USA, China, England, Canada, and Australia. Cluster 2 included Italy, German, France, Spain, Greece, and Turkey.

**Figure 3:**
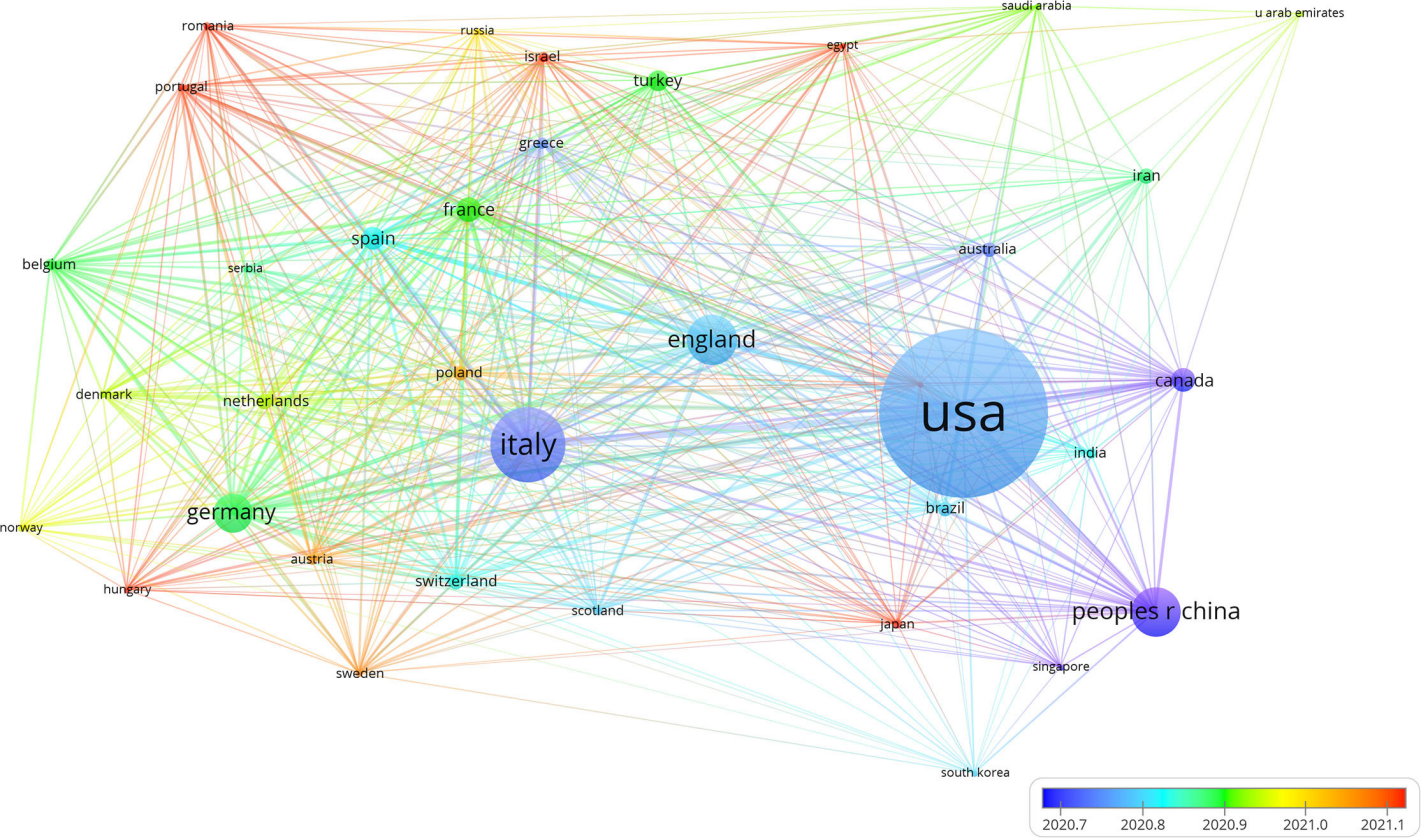
The timeline view of the country distribution. Different colors represented different years. The warmer the color was, the closer the year. The distribution of cardiac disorders also reflected the dynamic change of critically ill patients in different countries. China, Italy, Canada, Singapore, USA, and England mainly carried out research on the cardiac involvement of COVID-19 in the early stage. With the epidemic of COVID-19, Spain, Switzerland, France, Germany, Israel, Russia, and other countries also began to focus on the cardiac disorder in COVID-19.

All of the documents were from 102 countries and 4,020 organizations published before May 12, 2022. The countries that had published the most articles on COVID-19 with heart were the United States of America (USA) (*n* = 747, 36.9%), Italy (*n* = 324, 16%), and England (*n* = 213, 10.5%). This finding suggested that the study of COVID-19 influence on the heart in these countries may have played a critical role in cardiovascular research and USA was in a leading position in the field, which may benefit from the contributions of scientific research. The visualization map of the country was generated by the VOSviewer software. Each node represented a country, and the size of the node was proportional to the number of articles published. The lines between nodes represented cooperation between countries and denser lines corresponded to closer cooperation. These countries cooperated and exchanged with each other. They were mainly divided into two clusters as shown in Figure 2. Cluster 1 included the USA, China, England, Canada, and Australia who carried out research on the cardiac effects caused by COVID-19 in the early stage. Cluster 2 included Italy, German, France, Spain, Greece, and Turkey. As only critically ill patients were associated with cardiac involvement, the impact of COVID-19 on the heart can also reflect the changes in critically ill patients' distribution in different countries. With the progress of time, Spain, France, Germany, and other countries also began to focus on the cardiac disorder in COVID-19 (Figure 3).

### Distribution of Institutions

**Figure 4:**
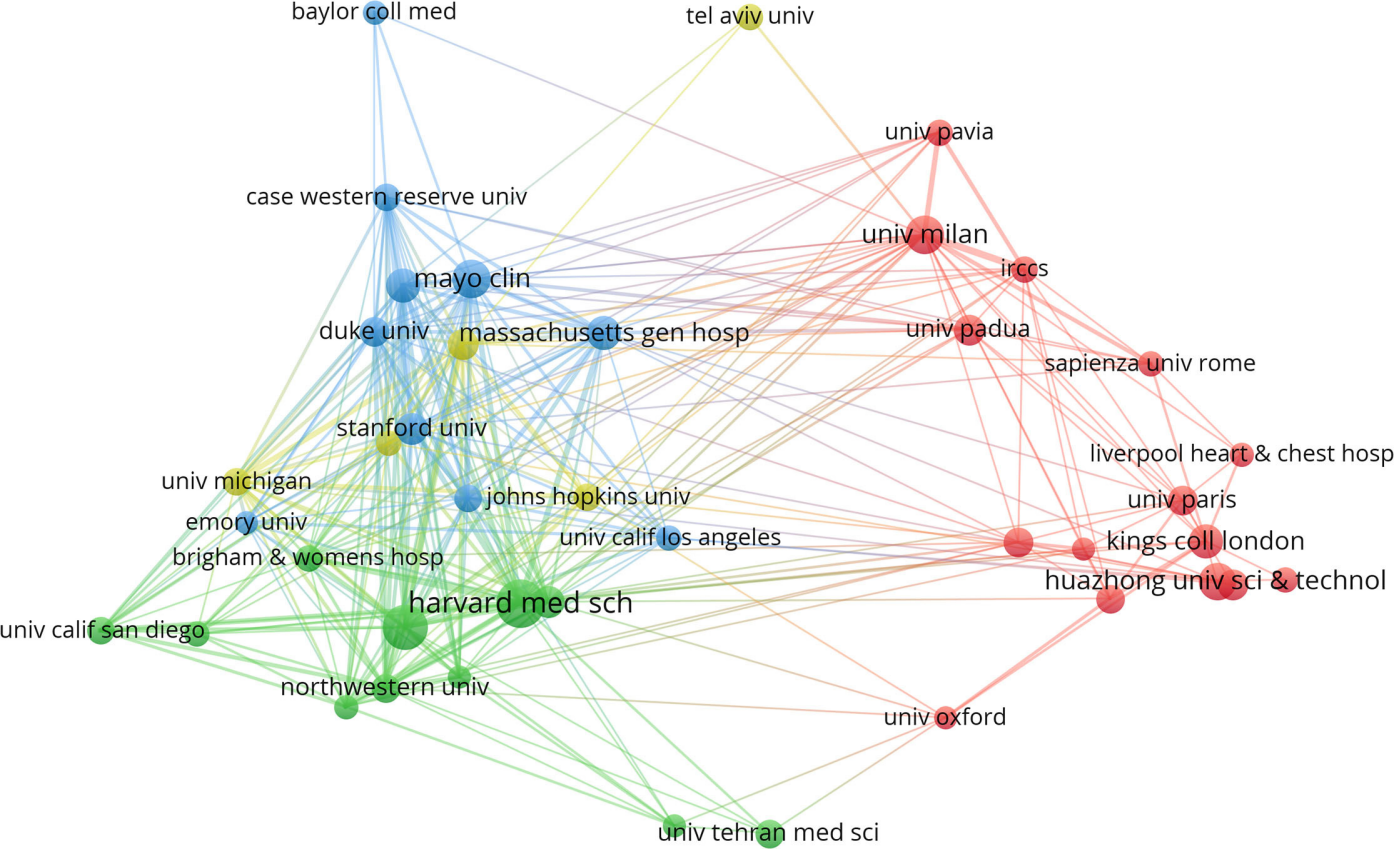
Detailed information of organization distribution. The size of the circle represented its number of publications. Harvard Medical School had published the highest number of research publications in this field followed by the Columbia University and Mayo Clinic. Various institutions cooperated with each other, however, mutual exchanges and cooperation were relatively limited. Harvard Medical School, Columbia University, and Stanford University cooperated with each other most frequently. Huazhong University of Science and Technology, King's College London, and Wuhan University cooperated more frequently.

The institution with the highest number of research publications in this field was the Harvard Medical School with a quantity of 67, followed by the Columbia University with a quantity of 56 and Mayo Clinic with a quantity of 43 (Table 2). Furthermore, we analyzed the cooperative relationships of major institutions and found that various institutions cooperated with each other. However, mutual exchanges and cooperation were relatively limited. Harvard Medical School, Columbia University, and Stanford University cooperated most frequently. Huazhong University of Science and Technology and King's College London and Wuhan University cooperated more frequently, which demonstrated that further cooperation was needed to a larger extent. Detailed information of organization distribution was shown in Figure 4. The research institutions with active cooperation were the University of Pittsburgh, Stanford University, Harvard University, Brigham and Women's Hospital, and Harvard Medical School.

**Table 2:** Detailed information of the top ten countries and organizations.

**Rank**	**Source**	**Publications**	**Citations**	**Rank**	**Source**	**Country**	**Publications**	**Citations**
1	USA	747	15,694	1	Harvard Medical School	USA	67	2,553
2	Italy	324	8,414	2	Columbia University	USA	56	1,270
3	England	213	3,997	3	Mayo Clinic	USA	43	1,704
4	China	211	10,409	4	University of Milan	Italy	42	1,483
5	Germany	165	3,440	5	Huazhong University of Science and Technology	China	39	1,952
6	France	104	2,048	6	Massachusetts General Hospital	USA	34	1,087
7	Canada	99	1,690	7	King's College London	England	33	384
8	Spain	96	1,173	8	Cleveland Clinic	USA	32	732
9	Turkey	87	612	9	Stanford University	USA	29	1,085
10	Netherlands	64	1,050	10	Icahn School of Medicine at Mount Sinai	USA	29	793

### Distribution of Authors

**Figure 5:**
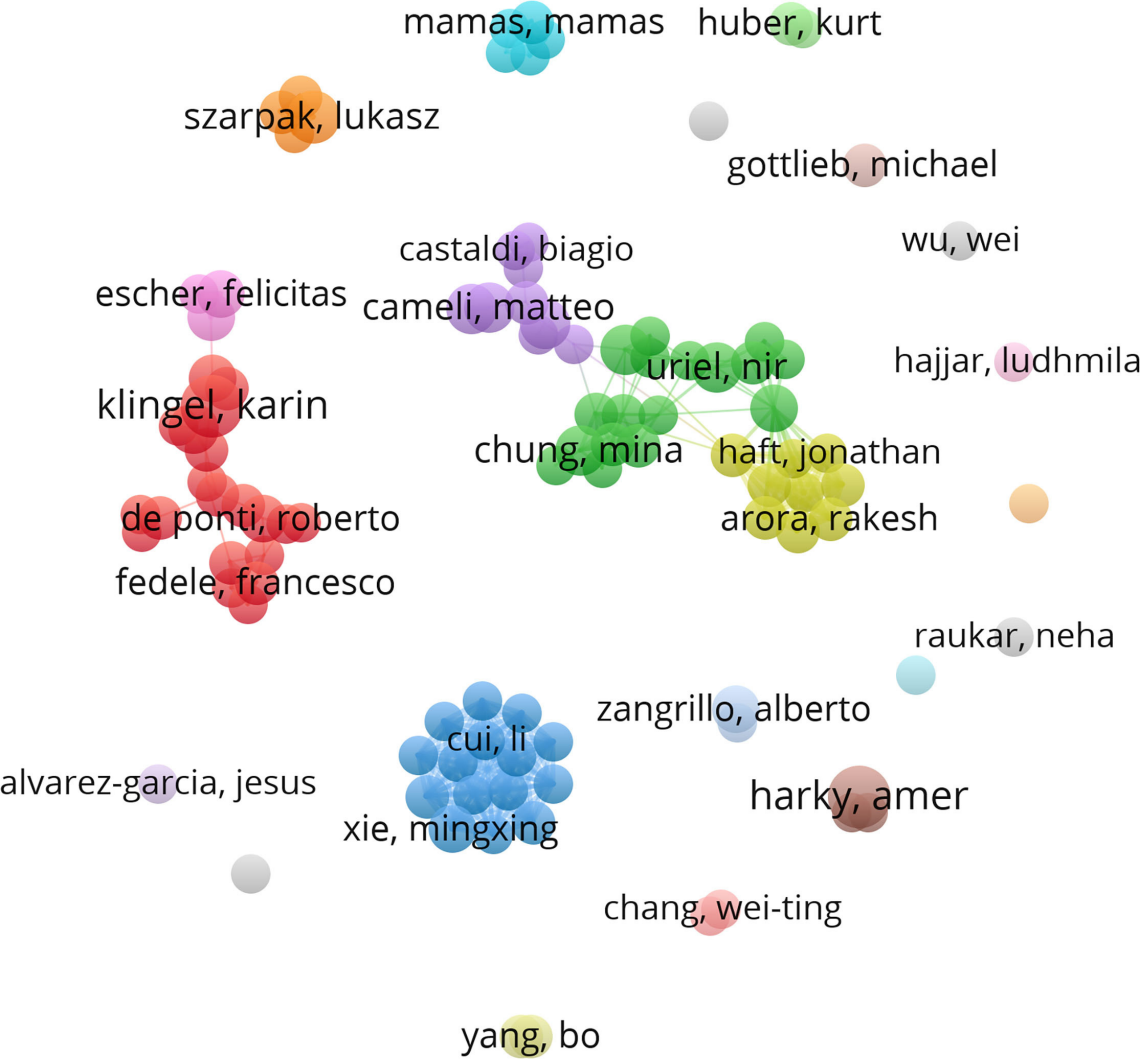
The detailed information of the authors' distribution. Each node represented an author, with larger nodes representing more published articles. Different colors referred to clusters of close cooperation. Karin Klingel and Amer Harky had the greatest number of published papers. Most authors were scattered and lacked stable and intensive cooperation and communication. Some authors mainly cooperated on a small scale in this research area. Karin Klingel only cooperated with DaoWen Wang, Enrico Ammirati, Burkert M. Pieske, and Carsten Tschoepe. Amer Harky only cooperated with Aung Oo and Ana Lopez-Marco.

A total of 13,739 authors published articles on COVID-19 in cardiac involvement (Table 3). Karin Klingel and Amer Harky from cardio-pathology, University Hospital Tuebingen, Germany, and the department of cardiothoracic surgery, Liverpool Heart and Chest, UK, had the greatest number of published papers (12, 0.6%), followed by Lukasz Szarpak (9, 0.4%), Matteo Cameli (8, 0.39%), Serafina Valente (8, 0.39%), Dao Wen Wang (8, 0.39%), and so on. Although the countries and institutions that published the most articles were almost from the USA, the authors with the largest number of articles were not mainly from the USA. The detailed information of the authors was shown in Figure 5. Each node represented an author, with larger nodes representing more published articles. Thicker lines implied closer cooperation between authors. Different colors referred to clusters of close cooperation. As shown in Figure 5, most authors were scattered and lacked stable and intensive cooperation. Some authors mainly communicated with each other on a small scale in this area. Karin Klingel, the author with the largest number of published articles, only cooperated with DaoWen Wang, Enrico Ammirati, Burkert M Pieske, and Carsten Tschoepe. Amer Harky only cooperated with Aung Oo and Ana Lopez-Marco. It was suggested that research on the cardiac disorder caused by COVID-19 was still relatively limited, lacking in-depth communication and cooperation.

**Table 3:** Detailed information of the top ten authors.

**Rank**	**Author**	**Country**	**Documents**	**Citations**	**Average citation/ Publication**
1	Karin Klingel	Germany	12	229	19.1
2	Amer Harky	England	12	187	15.6
3	Lukasz Szarpak	Poland	9	19	2.1
4	Matteo Cameli	Italy	8	54	6.8
5	Serafina Valente	Italy	8	56	7.0
6	Dao Wen Wang	China	8	1,092	136.5
7	Mina K Chung	USA	8	324	40.5
8	Ehtisham Mahmud	USA	8	235	29.4
9	Gianluca Pontone	Italy	8	140	17.5
10	Nir Uriel	USA	8	198	24.8

### Distribution of Journals

The top 10 journals published 361 articles, accounting for 17.8% of the total literature (Table 4). The Frontiers in Cardiovascular Medicine (65, 3.2%) had the highest number of outputs, followed by the ESC Heart Failure (59; 2.9%) and the Journal of Clinical Medicine (56; 2.8%). Most journals mainly belong to the cardiovascular field. Among the top 10 journals, Circulation and JAMA Cardiology had the highest impact factor, and more in-depth research was still needed in this field.

**Table 4:** Top 10 journals of COVID-19-mediated cardiac disorder.

**Rank**	**Source**	**IF**	**Publications**	**Citations**	**Average citation/ Publication**
1	Frontiers in Cardiovascular Medicine	6.050	65	184	2.8
2	Esc Heart Failure	4.411	59	442	7.5
3	Journal of Clinical Medicine	4.241	56	340	6.1
4	Journal of Cardiac Surgery	1.620	37	327	8.8
5	Cardiology in the Young	1.093	32	50	1.6
6	Journal of the American Heart Association	5.501	25	247	9.9
7	American Journal of Emergency Medicine	2.469	24	642	26.8
8	Scientific Reports	4.379	24	49	2
9	JAMA Cardiology	14.676	20	7,746	387.3
10	Circulation	29.690	19	2,217	116.7

### Citation Analysis/Co-cited Authors and Journals

**Figure 6:**
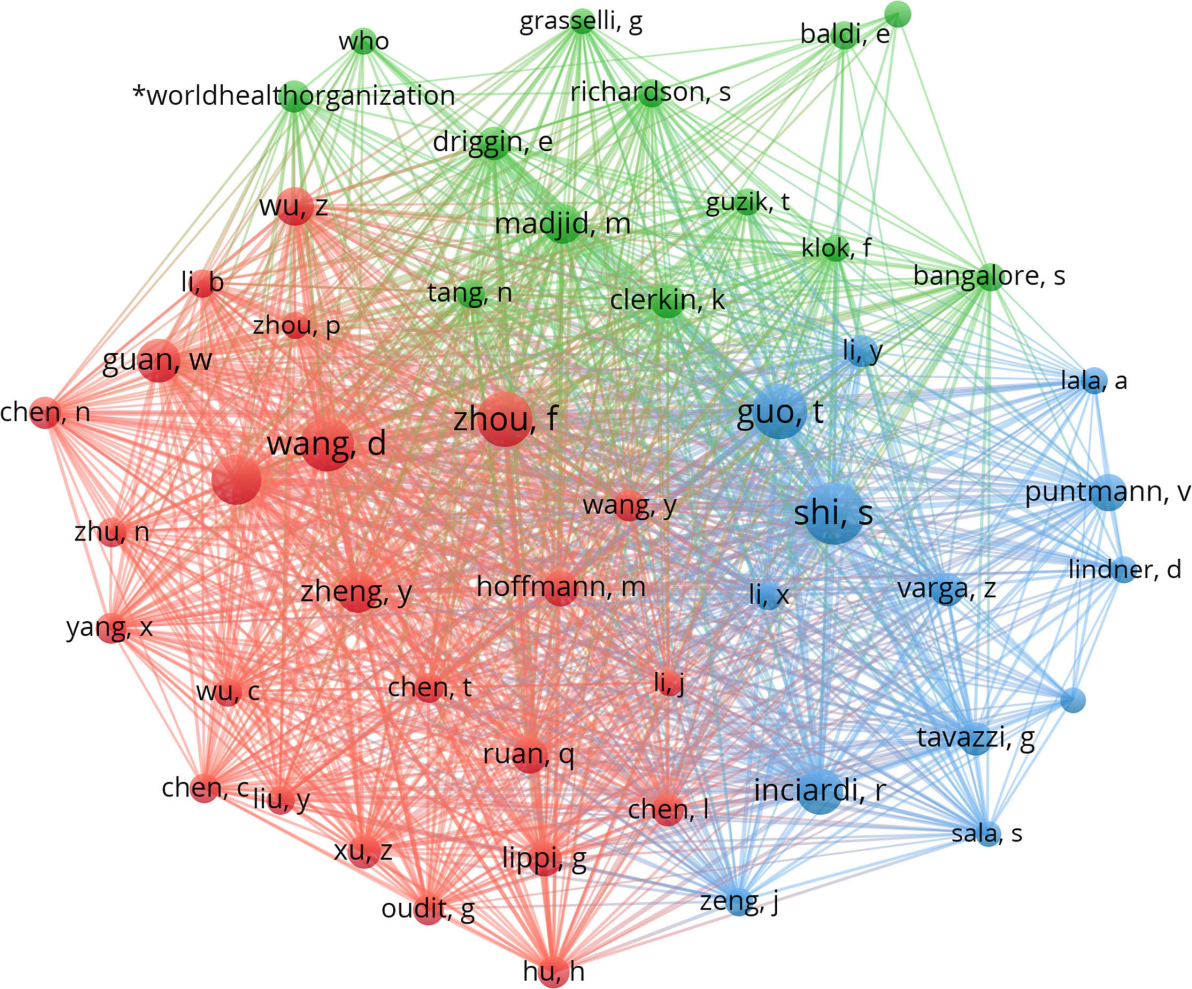
The distribution of all the co-cited authors. VOSviewer is a reliable tool for displaying the intellectual base and frontiers of a certain research field by performing co-citation analysis and burst detection. When two references were cited by the third reference at the same time, these two references constituted a co-citation relationship. The strength of the co-citation relationship between the two cited articles was proportional to the similarity of their research contents and the more times they were cited at the same time, the stronger the co-citation relationship was. There were two major clusters. Cluster 1 (blue) included Shaobo Shi, Tao Guo, Zsuzsanna Varga, and Riccardo M Inciardi; while cluster 2 (red) included Daowei Wang, Fei Zhou, Chaolin Huang, and Wei-Jie Guan. These authors occupied a pivotal position in this field.

Co-citation analysis is designed to measure the degree of relationship between articles. The influence of journals depends on the number of times they are co-cited, which reflects whether the journal has a significant influence in a particular research field. Among the 26,451 authors, 68 authors had been cited more than 100 times. Shaobo Shi from Renmin hospital of Wuhan University ranked as the first co-cited author with 665 citations followed by Fei Zhou from Chinese Academy of Medical Sciences, Peking Union Medical College, Beijing, China, and Tao Guo from the department of cardiology, Zhongnan Hospital of Wuhan University, Wuhan, China. Almost most of the top co-cited authors were from China and two were from the city of Wuhan. Moreover, all the co-cited authors often cooperated with each other from Figure 6. Among 7,563 co-cited journals, 7 journals were cited over 2,000 times. As shown in Table 5, the New England Journal of Medicine (3,390) was the most frequently cited journal, followed by Circulation (3,268) and JAMA Cardiology (2,814). Among the top 15 journals, the New England Journal of Medicine had the highest impact factor (IF) (91.245), followed by the Lancet with an IF of 79.321. According to the journal citation reports partition in 2021, all of the top 10 co-cited journals were distributed in the Q1 region.

**Table 5:** Top 10 co-cited authors and journals.

**Rank**	**Author**	**Citations**	**Country**	**Rank**	**Journal**	**IF (2021)**	**Citations**	**JCR (2021)**
1	Shaobo Shi	665	China	1	New England Journal of Medicine	91.245	3,390	Q1
2	Fei Zhou	537	China	2	Circulation	29.690	3,268	Q1
3	Tao Guo	533	China	3	JAMA Cardiology	14.676	2,814	Q1
4	Dawei Wang	511	China	4	Lancet	79.321	2,775	Q1
5	Chaolin Huang	446	China	5	Journal of the American College of Cardiology	24.094	2,482	Q1
6	Riccardo M Inciardi	376	Italy	6	European Heart Journal	29.983	2,472	Q1
7	Weijie Guan	337	China	7	JAMA-Journal of the American Medical Association	56.272	2,263	Q1
8	Yingying Zheng	290	China	8	Nature	49.962	727	Q1
9	Mohammad Madjid	276	USA	9	European Journal of Heart Failure	15.534	726	Q1
10	Zunyou Wu	256	China	10	Circulation Research	17.367	672	Q1

### Co-cited References and Top-Cited Articles

Co-citation analysis indicated that two references appeared in the reference list of a third citation article, and then the two references formed a co-citation relationship. We listed the top 10 most frequently cited references related to the research. Among the 39,581 co-cited references, 40 references were cited more than 100 times, and the references listed in the top three were all cited more than 500 times (Table 6). The most frequently cited reference topic was closely associated with cardiac injury and mortality in hospitalized COVID-19 patients in Wuhan, China. Almost all the co-cited literature of COVID-19 combined with cardiac injury were mainly from the year 2020 and was reported by articles and case reports. It was suggested that articles published in the early stage of the epidemic deserved high citations.

**Table 6:** Top 20 co-cited articles.

**Rank**	**Articles**	**Author**	**Journal**	**Year**	**Type**	**Occurrences**	**Total link strength**
1	Association of Cardiac Injury With Mortality in Hospitalized Patients With COVID-19 in Wuhan, China	Shaobo Shi	JAMA Cardiology	2020	Article	559	2,802
2	Clinical course and risk factors for mortality of adult inpatients with COVID-19 in Wuhan, China: a retrospective cohort study	Fei Zhou	Lancet	2020	Article	532	2,790
3	Cardiovascular Implications of Fatal Outcomes of Patients With Coronavirus Disease 2019 (COVID-19)	Tao Guo	JAMA Cardiology	2020	Article	524	2716
4	Clinical features of patients infected with 2019 novel coronavirus in Wuhan, China	Chaolin Huang	Lancet	2020	Article	376	2,113
5	Clinical Characteristics of 138 Hospitalized Patients With 2019 Novel Coronavirus-Infected Pneumonia in Wuhan, China	Dawei Wang	JAMA	2020	Article	337	1,980
6	Cardiac Involvement in a Patient With Coronavirus Disease 2019 (COVID-19)	Riccardo M Inciardi	JAMA Cardiology	2020	Case Report	289	1,717
7	Clinical Characteristics of Coronavirus Disease 2019 in China	Wei-Jie Guan	New England Journal of Medicine	2020	Article	275	1,468
8	COVID-19 and the cardiovascular system	Ying-Ying Zheng	Nature Reviews Cardiology	2020	comment	271	1,530
9	Characteristics of and Important Lessons From the Coronavirus Disease 2019 (COVID-19) Outbreak in China: Summary of a Report of 72 314 Cases From the Chinese Center for Disease Control and Prevention	Zunyou Wu	JAMA	2020	Article	245	1,302
10	Outcomes of Cardiovascular Magnetic Resonance Imaging in Patients Recently Recovered From Coronavirus Disease 2019 (COVID-19)	Valentina O Puntmann	JAMA Cardiology	2020	Article	233	892
11	Clinical predictors of mortality due to COVID-19 based on an analysis of data of 150 patients from Wuhan, China	Qiurong Ruan	Intensive Care Medicine	2020	Letter	217	1,437
12	SARS-CoV-2 Cell Entry Depends on ACE2 and TMPRSS2 and Is Blocked by a Clinically Proven Protease Inhibitor	Markus Hoffmann	Cell	2020	Article	205	1,223
13	COVID-19 and cardiovascular disease	Kevin J. Clerkin	Circulation	2020	Review	202	1,072
14	Cardiovascular Considerations for Patients, Health Care Workers, and Health Systems During the COVID-19 Pandemic	ElissaDriggin	Journal of the American College of Cardiology	2020	Review	197	1,127
15	Myocardial localization of coronavirus in COVID-19 cardiogenic shock	Guido Tavazzi	European Journal of Heart Failure	2020	Case Report	195	1,164
16	Pathological findings of COVID-19 associated with acute respiratory distress syndrome	Zhe Xu	Lancet Respiratory Medicine	2020	Case Report	191	1,223
17	Endothelial cell infection and endotheliitis in COVID-19	Zsuzsanna Varga	Lancet	2020	Case Report	179	990
18	Potential Effects of Coronaviruses on the Cardiovascular System	Mohammad Madjid	JAMA Cardiology	2020	Review	177	934
19	Epidemiological and clinical characteristics of 99 cases of 2019 novel coronavirus pneumonia in Wuhan, China: a descriptive study	Nanshan Chen	Lancet	2020	Article	172	1,005
20	A Novel Coronavirus from Patients with Pneumonia in China, 2019	Na Zhu	New England Journal of Medicine	2020	Article	158	763

### Analysis of Hotspots and Main Research Directions

**Figure 7:**
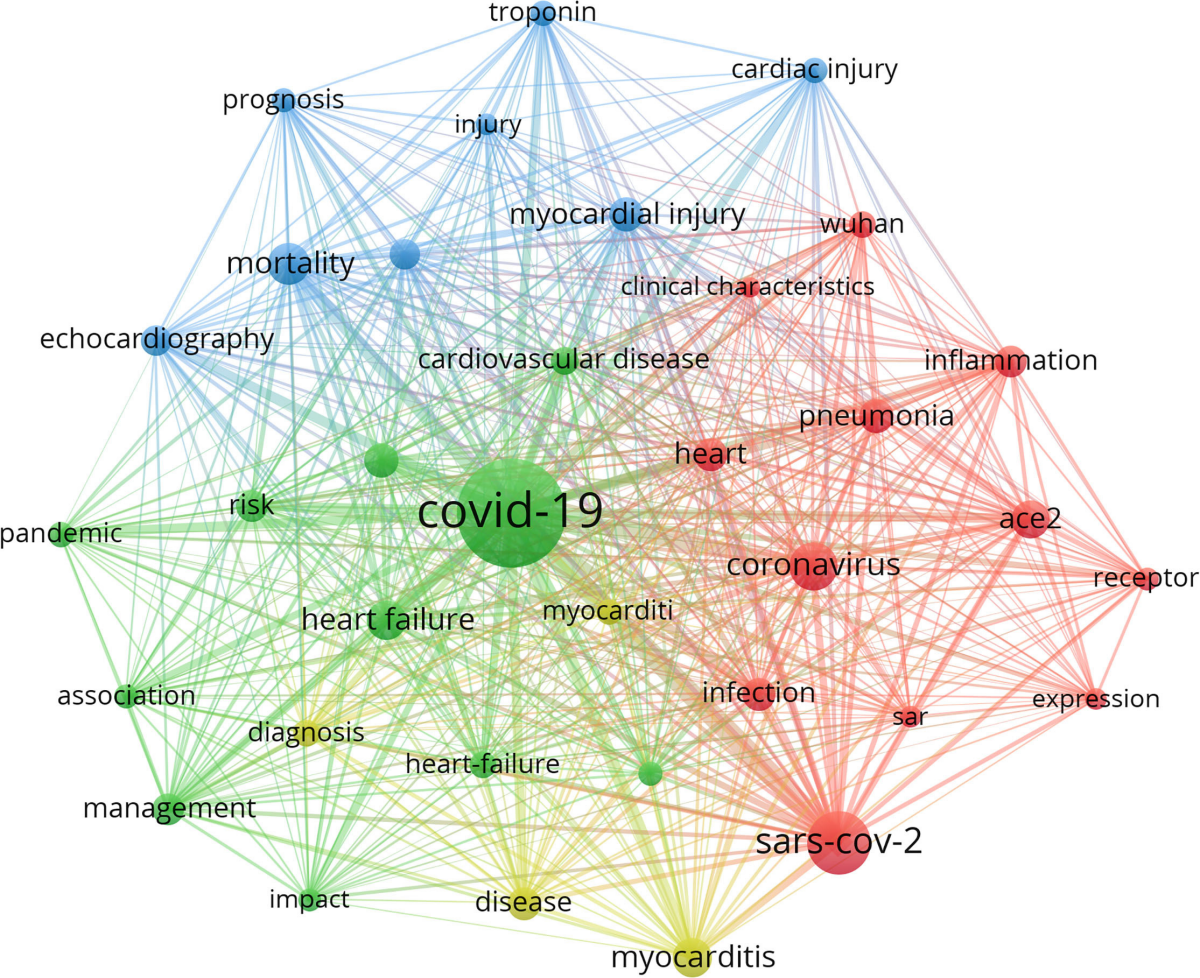
The network mapping on keywords of cardiac involvement caused by COVID-19. The size of each circle represented the weight of a keyword. The distance between the two circles indicated the relatedness between the two circles. The stronger the relatedness, the shorter the distance. The color of the circles represented the respective cluster class. This figure displayed three clusters of red, blue, and green, indicating three research directions. Red clusters were composed of SARS-CoV2, coronavirus, ace2, pneumonia, inflammation, Wuhan, and heart. The keywords of the green cluster were COVID-19, management, heart failure, pandemic, and cardiovascular disease. The keywords of the blue cluster were myocardial injury, mortality, echocardiography, cardiac injury, and troponin.

**Figure 8:**
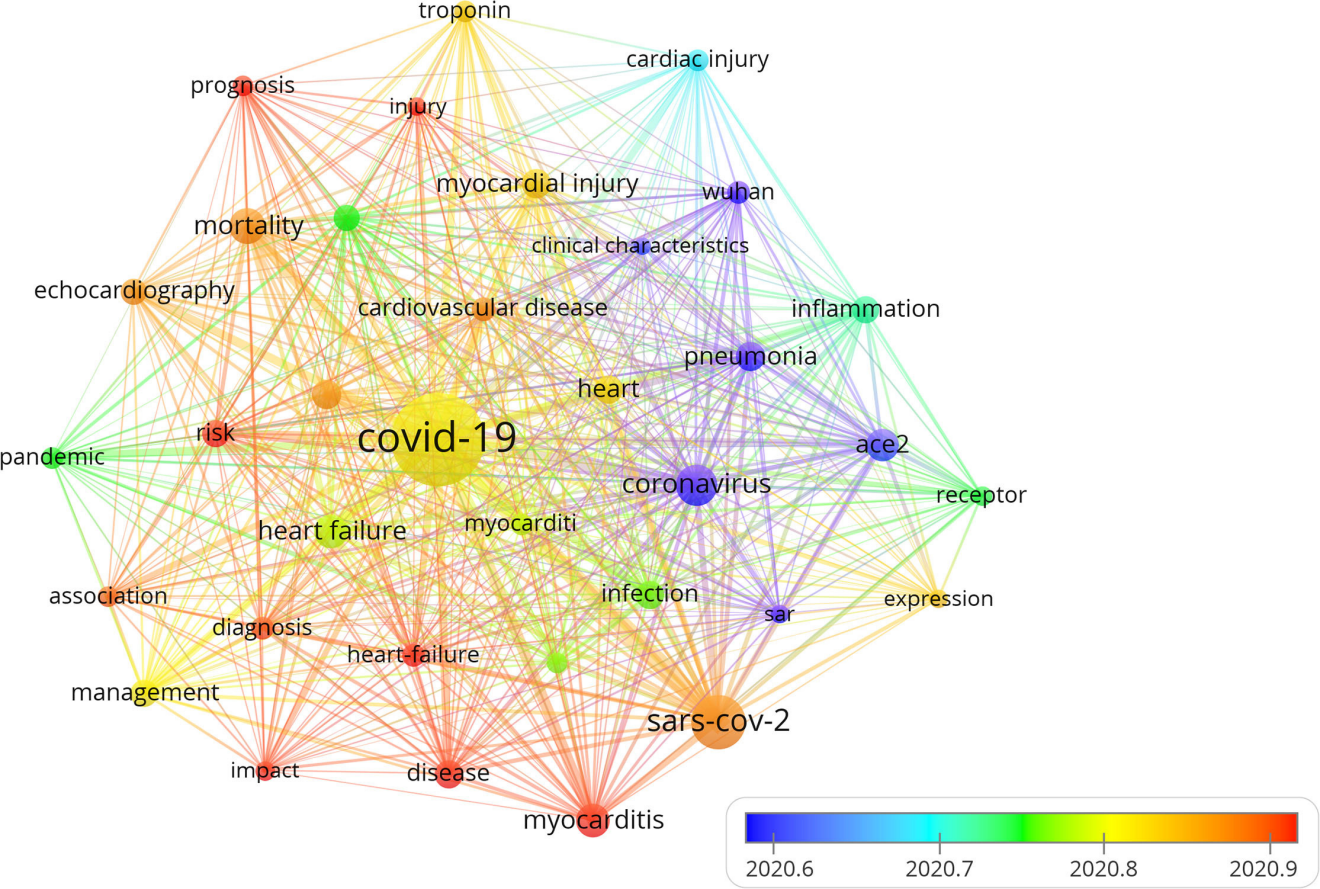
The timeline visualization map of keywords clustering analysis on cardiac involvement in COVID-19. Different colors represented different years. The warmer the color was, the closer the year. This figure displayed a timeline chart of COVID-19-mediated cardiac involvement based on VOSviewer software. The figure visually reflected the phased hotspots and epidemic status of severe COVID-19 from the time dimension. The initial research mainly focused on pneumonia caused by coronavirus through the receptor of ACE2 in Wuhan. With the spread of the disease, it was found that severe pneumonia infection with an obvious inflammatory response mediated by SARS-CoV-2 can lead to cardiac injury. With the global pandemic of COVID-19, the keywords referring to myocadiac injury, including HF, myocarditis, and troponin had become increasingly prominent. With the deepening understanding of the disease, further identification of HF and myocarditis through echocardiography was of great significance for the prognosis in severe COVID-19 patients.

Keywords summarize the research topics. Through the analysis of keywords, we can understand the research hotspots in specific fields. Table 7 displayed the high-frequency keywords. Among these keywords, myocarditis, heart failure (HF), and myocardial injury ranked as the top three cardiac injury terms, suggesting that COVID-19 had a substantial effect on cardiac function. We used the VOSviewer software to cluster the keywords. The circle and label form an element, the color identified the cluster to which it belongs. Figure 7 displayed the clusters of red, blue, and green, indicating three research directions. Red clusters were composed of SARS-CoV-2, coronavirus, ace2, pneumonia, inflammation, Wuhan, and heart. The keywords of the green cluster were COVID-19, management, heart failure, pandemic, and cardiovascular disease. The keywords of the blue cluster were myocardial injury, mortality, echocardiography, cardiac injury, and troponin. The timeline view was designed based on the interaction and mutual relationship between keywords in a particular field, and it was used to explore the evolutionary track and stage characteristics in the field. Figure 8 displayed a timeline chart of COVID-19 mediated cardiac involvement based on VOSviewer software; it visually reflected the phased hotspots and epidemic status of severe COVID-19 from the time dimension. The initial research mainly focused on pneumonia caused by SARS-CoV2 through ACE2 receptor in Wuhan. With the progress of the disease, it was found that severe pneumonia with an obvious inflammatory response mediated by SARS-CoV2 can cause cardiac injury. With the global pandemic of COVID-19, the keywords referring to myocardiac injury, including HF, myocarditis, and troponin had become increasingly prominent. With further understanding of the disease, identification of HF and myocarditis through echocardiography was of great significance for the diagnosis and prognosis in severe COVID-19 patients. As only severe patients were complicated with heart injury, the situation of severe patients can also be estimated by cardiac injury. The timeline view also reflected the fluctuations in patients with severe pneumonia in various countries to some extents.

**Table 7:** Top 20 keywords related to COVID-19-mediated cardiac involvement.

**Rank**	**keyword**	**Occurrences**	**Total link strength**	**Rank**	**Keyword**	**Occurrences**	**Total link strength**
1	COVID-19	1,297	2,034	11	Heart	123	312
2	SARS-CoV-2	434	1,008	12	Disease	119	294
3	Coronavirus	255	637	13	Infection	118	358
4	Mortality	197	427	14	Inflammation	116	323
5	Myocarditis	175	421	15	Management	116	263
6	Heart failure	170	385	16	Risk	116	250
7	Ace2	157	450	17	Coronavirus disease 2019	108	196
8	Myocardial injury	128	385	18	Echocardiography	100	193
9	Pneumonia	127	364	19	Cardiovascular disease	89	227
10	Outcome	127	325	20	Heart-failure	83	202

## Discussion

COVID-19 caused by the SARS-CoV-2 was reported in Wuhan, China, in December 2019 and had spread across the whole globe and adversely affected the livelihood of millions of people (10). Previous studies had demonstrated that a substantial majority of patients hospitalized developed an acute COVID-19 cardiovascular syndrome, which manifested with a variety of clinical presentations ranging from acute cardiac injury with cardiomyopathy, ventricular arrhythmias, and hemodynamic instability in the absence of obstructive coronary artery disease (11). Early studies had also shown that almost all patients with severe illness had severe myocardial damage, which almost reached 100% in critically ill patients and 70–80% in severely ill patients. However, the specific role of cardiac involvement in COVID-19 had not been elucidated yet. Bibliometric can not only offer a quantitative and statistical analysis of publications in specific fields but also accurately reflect the most representative studies (12, 13). In addition, by presenting numerous data in the form of knowledge maps, researchers can comprehensively analyze the development of a discipline and understand the frontier trends.

Our study discovered that about half of the countries and regions in the world had reported SARS-CoV-2 combined with the cardiac disorder, suggesting that cardiac involvement caused by severe COVID-19 was not uncommon. The USA was the country that had published the most articles on SARS-CoV-2 combined with cardiac in the world, which was almost corresponding to the high mortality in America according to the world health organization (https://covid19.who.int/?mapFilter=deaths). Italy and England ranked as the second and third countries in the number of articles which was also consistent with the order of the mortality rate in Europe by the world health organization (WHO). In addition to the country with the most published articles, the scientific research institutions or organizations with the most published articles were also mainly from the USA. However, Karin Klingel and Amer Harky, the authors with the most published articles were not from the USA. Karin Klingel mainly majored in SARC-CoV-2 mediated myocarditis, while Amer Harky worked as a surgeon and majored in cardiac surgery which suggested that COVID-19 had also a significant influence on cardiac surgery application. Although some scholars had cooperation to some extents, most cooperation was relatively limited and require greater and deeper improvement. Moreover, although many literature had reported that COVID-19 was complicated by heart injury, most of the articles belong to journals with a medium level of impact factor. The main reason may due to that most scholar mainly focused on the lung damage caused by SARC-CoV-2 and the number of severe patients may decrease with the continuous variation of the virus and the development of vaccines and anti-viral drugs.

Co-citation analysis can demonstrate the weight of authors and journals in a specific research field. Among the 26,451 co-cited authors, 68 authors had been cited more than 100 times. Shaobo Shi from Renmin hospital of Wuhan University ranked as the first co-cited author with 665 citations followed by Fei Zhou from the Chinese Academy of Medical Sciences, Peking Union Medical College, China, and Tao Guo from the department of cardiology, Zhongnan Hospital of Wuhan University, China. Shaobo Shi had demonstrated that cardiac injury was a common phenomenon among hospitalized patients with COVID-19 in Wuhan, and it was closely associated with a higher risk of mortality (14), while Fei Zhou had demonstrated that the potential risk factors of older age, high sequential organ failure assessment (SOFA) score, and d-dimer could predict patients with poor prognosis. Prolonged viral shedding offered the rationale strategy of isolation of infected patients and proper antiviral interventions (15). The third co-cited author Tao Guo discovered that myocardial injury was significantly associated with the fatal outcome of COVID-19, while the prognosis of patients without underlying cardiovascular diseases was relatively favorable. Myocardial injury was associated with cardiac dysfunction and arrhythmias. Aggressive treatment may be considered for patients at high risk of myocardial injury (16). All the three studies were clinical studies from China, which respectively elaborated the impact of SARS-CoV2 from different aspects and heart damage caused by SARS-CoV2 in the early stage. The three articles had been published in international top journals, which were worthy of high reference.

The presentation and distribution of keywords and hotspots can help us quickly identify the frontier and directions of a certain research field. After capturing the hot spots and forewords of SARS-CoV2-mediated cardiac injury, we found that myocarditis and HF were the main complications of SARS-CoV2 mediated heart injury. Previous literature also reported that myocarditis and HF were also one of the main reasons for death in severe patients.

### Myocarditis

Although myocardial injury occurred in 20–30% of hospitalized patients with COVID-19 infection, cardiovascular complications contributed to approximately 40% of all COVID-19-related deaths according to a previous study (17). SARS-CoV-2-mediated myocarditis ranged from ordinary myocarditis with slightly elevated myocardial enzymes to severe myocarditis accompanied by hemodynamic changes, HF, and even cardia shock. Most cases of myocarditis related to COVID-19 infection occurred in the initial phase of infection and were self-limited. The risk of death was significantly increased in patients with severe myocarditis. In the early stage, many studies had reported the injury of myocarditis mediated by SARS-CoV-2 (18, 19). Although the virus continued to mutate and its virulence decreased with the virus mutation, there were still reports of scattered severe myocarditis. Clinical myocarditis during the acute phase of illness had been reported in only 1.4–7.2% of cases in autopsy studies (20). Delayed acute myocarditis with COVID-19 infection had also been reported recently. Alistair Thomson had recently reported a 39-year-old female with no significant previous medical history and confirmed delta variant COVID-19 infection. Endomyocardial biopsy discovered diffuse interstitial macrophage infiltration and small vessel thrombosis. Despite treatment with tocilizumab, high-dose steroids, and intravenous immunoglobulin, she eventually died due to disease-related complications (21). Although myocarditis was mainly secondary to acute inflammatory disease of the lung, approximately 60% to 80% of patients who recovered from COVID-19 were found evidence of myocarditis by cardiac magnetic resonance imaging at a median of 70 days from infection (22). Mahmoud Ismayl described a case of delayed-onset fulminant myocarditis that developed 5 weeks after mild COVID-19 infection resulting in cardiogenic shock and the need for mechanical circulatory support (23). The direct infiltration of SARS-CoV-2 and the infiltration of immune cells mediated by systemic inflammatory response were the main pathogenesis of viral myocarditis. The incidence of acute and delayed acute myocarditis was consistent with the study that SARS-CoV-2 may be associated with a postinfectious, immune-mediated myopathy (24). Another COVID-19-associated myocarditis was virus vaccine-mediated myocarditis. Myocarditis following mRNA COVID-19 vaccination predominantly occurred among young males in their teens or twenties a few days later of the second dose of the vaccine (25). According to the US Centers for Disease Control and Prevention, myocarditis/pericarditis rates were almost 12.6 cases per million after second-dose mRNA vaccine among individuals 12–39 years of age (26). Almost all the clinical symptoms were mild, and this young population exhibited a good prognosis.

### Heart Failure

Another hotpot related to COVID-19-associated cardiac dysfunction was HF. HF was a common disease state that can be encountered at different stages during COVID-19. New or pre-existing HF in the setting of COVID-19 can present challenges that can be encountered in presentation, management, and prognosis. Lessons from the previous coronavirus and influenza epidemics implied that viral infections can exacerbate a pre-existing HF, with multiple studies showing increased mortality during influenza-like illness seasons (27). With the more aggressive COVID-19 infection, HF patients were considered at a higher risk of acute deterioration, and multiple mechanisms may be responsible for triggering and aggravating this process. It was also reported that the virus almost led to 15–29% kidney impairment in COVID-19 patients (28), which may result in volume overload that may exacerbate a pre-existing chronic HF. Instead of aggravating the pre-existing cardiac disease, new onset of HF was observed in a quarter of hospitalized COVID-19 patients and as much as one-third of those patients admitted to the intensive care unit (ICU) did not have a history of HF (29).

Acute HF was suspected to be a direct consequence of COVID-19, with a dramatic impact on mortality. During COVID-19 hospitalization, about one-third of patients with previous HF had an acute decompensation of HF (30); however, acute HF can be triggered not only as decompensation of chronic HF but also as a new-onset HF (31). In an Italian multicenter study, acute HF occurred in 9.1% of patients during hospitalization, and almost half of them were new-onset HF with no previous HF history (30). The main reasons for COVID-19-induced HF included virus directly induced infiltration of inflammatory cells, system pro-inflammatory cytokines releasing syndrome, endothelial injury coupled with micro-thrombosis, and ARDS or respiratory failure that could lead to HF due to severe hypoxia (32). In another study of 131 patients who died of COVID-19, 49% of all deaths were attributed to HF in patients without a previous history of cardiovascular disease (33). We can speculate that respiratory failure including ARDS, cardiac injury, and HF were the most common sequelae of COVID-19. Since SARS-CoV2 was still evolving, the extent of the degree of cardiac involvement was still elusive.

In COVID-19 patients presenting acute HF, left ventricular (LV) systolic function was not usually reported; on the contrary, impairment of right ventricular (RV) systolic function and LV diastolic function can be detected more commonly (34). In one study of 100 patients hospitalized for COVID-19, 32% were reported to have normal echocardiography, whereas 39% presented RV dilatation and dysfunction and 16% LV diastolic dysfunction, while less than 10% of patients were reported with LV ejection fraction disorder (35). Accordingly, LV diastolic impairment with elevated LV filling pressures (E/e' ratio) could be observed in a quarter of patients with COVID-19. Consistently, patients hospitalized with COVID-19 showed a high likelihood of preserved ejection fraction (HFpEF) as compared with patients without COVID-19 according to the score of the Heart Failure Association (HFA) of the European Society of Cardiology (ESC), and HFpEF was regarded as the main cardiac structural and functional alterations and myocardial injury (36).

The link between COVID-19 and HF was complex. First of all, the COVID-19 pandemic had an obvious impact on HF management and increased mortality due to HF had been shown during the pandemic. Several studies showed a reduction in HF hospitalizations, ranging from 30 to 66% in different countries. Second, pre-existing HF was a risk factor for a more severe condition of clinical course in COVID-19 and an independent predictor of in-hospital mortality. Patients with a history of chronic HF were prone to develop into acute decompensated HF after a COVID-19 diagnosis (37). Third, patients hospitalized for COVID-19 may aggravate into acute decompensation of chronic HF and *de-novo* HF as a consequence of myocardial injury. In a word, HF was closely associated with cardiac injury in COVID-19 and deserved further study.

### Limitation

VOSviewer cannot fully represent the original distribution and wholly replace system retrieval. The uneven quality of collected literature data and cumbersome document screening process can result in reduced credibility of atlas drawing. The constant updates of data also lead to the retrieval results different from the actual number of included articles. Therefore, a more accurate literature analysis should be based on the knowledge map constructed by the VOSviewer combined with specific literature. Nevertheless, literature analysis based on visualization also helps the scholars quickly understand the research hotspots and development trends of COVID-19 in cardiovascular science to some extents.

## Conclusion

The research on the cross-talk between COVID-19 and cardiac involvement revealed that cardiac disorder was common in the world, and the USA, Italy, England, and China were the leading countries in this research by using the VOSviewer software for visual analysis. Among the research organization, Harvard Medical School was the institution with the highest influence on achievements. Different countries and institutions need to strengthen cooperation and exchange with each other. At present, the research on COVID-19-related cardiac disorders should concentrate on myocarditis and HF, especially left ventricular diastolic dysfunction and right ventricular systolic dysfunction which will also be the focus of future research.

## Data Availability Statement

The raw data supporting the conclusions of this article will be made available by the authors, without undue reservation.

## Author Contributions

SX, WW, and SZ designed the research. SX and XZ collected the primary data. XZ and HX preliminary screened out the irrelevant literature. SX, XZ, and HX checked and sorted out the literature again and analyzed the data. SX wrote the primary manuscript. WW and SZ wrote and revised the final manuscript. All authors listed have read and approved it for publication.

## Funding

This work was supported by the Chinese Academy of Medical Sciences (CAMS), the Innovation Fund for Medical Sciences (CIFMS) (2021-I2M-1-003), and the CAMS Endowment Fund (2021-CAMS-JZ004).

## Conflict of Interest

The authors declare that the research was conducted in the absence of any commercial or financial relationships that could be construed as a potential conflict of interest.

## Publisher's Note

All claims expressed in this article are solely those of the authors and do not necessarily represent those of their affiliated organizations, or those of the publisher, the editors and the reviewers. Any product that may be evaluated in this article, or claim that may be made by its manufacturer, is not guaranteed or endorsed by the publisher.
